# Recursive Alterations of the Relationship between Simple Membrane Geometry and Insertion of Amphiphilic Motifs

**DOI:** 10.3390/membranes7010006

**Published:** 2017-02-08

**Authors:** Kenneth Lindegaard Madsen, Rasmus Herlo

**Affiliations:** Molecular Neuropharmacology and Genetics Laboratory, Department of Neuroscience and Pharmacology, Faculty of Health and Medical Sciences, Panum Institute 18.6, University of Copenhagen, Copenhagen N DK-2200, Denmark

**Keywords:** amphipathic helix, BAR domain, membrane curvature, curvature sensing, membrane scission, lipid packing defect, surface tension, GUV, liposomes

## Abstract

The shape and composition of a membrane directly regulate the localization, activity, and signaling properties of membrane associated proteins. Proteins that both sense and generate membrane curvature, e.g., through amphiphilic insertion motifs, potentially engage in recursive binding dynamics, where the recruitment of the protein itself changes the properties of the membrane substrate. Simple geometric models of membrane curvature interactions already provide prediction tools for experimental observations, however these models are treating curvature sensing and generation as separated phenomena. Here, we outline a model that applies both geometric and basic thermodynamic considerations. This model allows us to predict the consequences of recursive properties in such interaction schemes and thereby integrate the membrane as a dynamic substrate. We use this combined model to hypothesize the origin and properties of tubular carrier systems observed in cells. Furthermore, we pinpoint the coupling to a membrane reservoir as a factor that influences the membrane curvature sensing and generation properties of local curvatures in the cell in line with classic determinants such as lipid composition and membrane geometry.

## 1. Introduction

The cellular membrane is not merely an inert platform for cellular processes, but it also actively regulates the localization, activity, and signaling properties of different types of proteins through geometric and/or compositional cues [1,2,3]. Some of the proteins that are recruited to the membrane in turn modulate membrane morphology, as in the case of Bin/amphiphysin/Rvs (BAR) domain proteins and dynamin [4,5], and further protein recruitment will depend on the interaction between the protein and membrane in a recursive manner. Simple geometric models do not integrate the membrane as a dynamic substrate and are therefore inadequate to explain such recursive phenomena.

**Figure 1:**
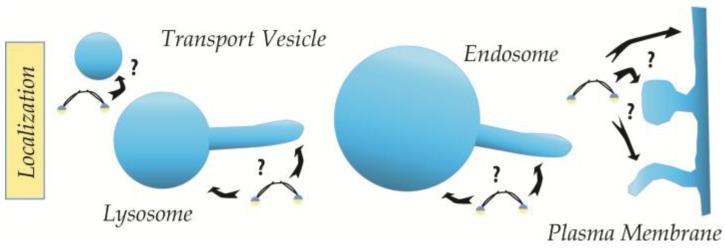
Different membrane geometries recruiting intracellular proteins. Protein recruitment to different membrane compartments with different local curvatures are exemplified here with N-BAR (BAR domains containing N-terminal amphipathic helices) proteins containing crescent shaped BAR domain dimers and amphipathic helices (**blue**/**yellow**). Arrows indicate potential sites of protein-membrane interactions.

The membrane morphology throughout the cell varies greatly, and trafficking processes, in particular, involve dynamic regulation of morphology in the shape of tubules and vesicles [6]. Moreover, these cellular membranes are not uniform entities with homogenously distributed lipids throughout the cell, but rather contain specific compartments, and even sub-compartmental regions and domains, which are characterized by different lipid compositions [7]. Localization to specific lipid compositions can be mediated by interaction between protein domains, such as Phox (PX) and the Pleckstrin Homology (PH), and specific lipid headgroups [8], or via electrostatic interactions, e.g., between negatively charged lipids and BAR domains. These domains also show high specificity for certain membrane geometries that fit their inherent crescent shape [9,10,11] (see Figure 1).

In addition to adsorptive binding regimes, selective recognition and stabilization of membrane geometries and compositions can be obtained through amphiphilic insertion motifs (AIMs), which are motifs that contain a hydrophilic part opposing a hydrophobic part [12,13]. These motifs interact with a membrane by inserting asymmetrically into one leaflet of the membrane, and are thus dependent on the presence of transient lipid packing defects (LPDs), which generate binding sites for AIM by disclosing the hydrophobic interior of the membrane. Unsaturation of lipid side-chains can promote formation of LPDs by inhibiting the close packing otherwise seen with cylindrical saturated lipids [14,15], and thereby provide compositional cues for interaction. The prevalence of these LPDs also correlates directly with the geometric curvature of the membrane, and different geometric models of curvature-sensitive protein recruitment of AIMs have consequently been suggested [13,16,17]. Common among these models are a continuous membrane curvature sensing (MCS), where the density of protein binding continuously increases with higher curvatures (or higher abundance of unsaturated lipids).

An intimate relation between the size of the AIM and such continuous MCS regime has been observed in vitro [18], and recently a study elegantly showed that the in vitro dependence of an AIM on LPDs was recaptured in cells, and could be modulated by changing either the lipid composition or the geometrical curvature of liposomes [19]. In addition to sensing of the curvature, AIMs are also capable of inducing local curvatures in membranes [20,21,22,23], and a direct relationship between the effective size of the AIM and the membrane curvature generation (MCG) property has been hinted experimentally [24]. AIMs, being both MCS and MCG, therefore serve as good model systems for recursive binding regimes with membranes. However, because the current geometric models of MCS do not integrate the membrane as a dynamic substrate, they do not provide explanation for phenomena, where a regulated recruitment of proteins modulate the membrane, e.g., during tubulation and fission. In addition, the simple geometric models do not explain discrepancies in experimental observations regarding the MCS of lipids between in vitro assays that utilize small unilamellar vesicles (SUVs) and assays that utilize the tubes pulled from giant unilamellar vesicles (GUVs). Such discrepancies have been suggested to be rooted in differential coupling to lipid reservoirs [25], however the theoretical framework for this contributing factor has yet to be addressed.

Here we outline a transition from a simple geometrical model of insertion of AIMs into a model that combines simple geometry and basic thermodynamics. By treating membranes as fluent entities rather than static platforms, we allow for alterations of the intrinsic properties along the membrane surfaces. This model allows the integration of recursive changes of the membrane substrate during saturation of binding and underlines the intimate connection between the MCS and MCG properties. Furthermore, it illustrates the importance of considering the degree of coupling to a membrane reservoir when predicting the local properties of a membrane substrate. In addition, the degree of coupling has important implications for fission processes from closed, as opposed to open, membrane compartments. Ultimately, membrane reservoir coupling emerges as an additional qualitative aspect of membrane identity in line with lipid composition and membrane geometry.

## 2. Different Modes of Interaction

**Figure 2:**
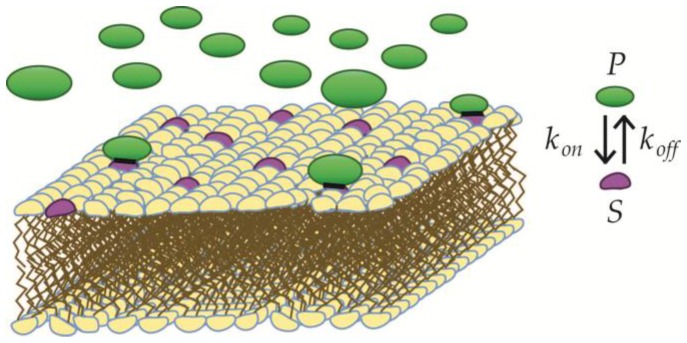
Adsorptive binding of protein to independent binding sites. Adsorptive binding of proteins (**green**) to specific binding sites (**purple**) in the membrane is illustrated. The binding sites are assumed to be independent, and the binding is characterized by an on-rate (*k*_on_) and an off-rate (*k*_off_).

In adsorptive binding regimes (see Figure 2), e.g., the electrostatic interactions between membrane and BAR domains [26,27], the amount of bound protein (*P_bound_*) to the binding sites (*S*) can be deduced from the law of mass action, if the binding sites can be assumed to be mutually independent, (see Equation (1)).

(1) $$\left[P_{bound}\right]=\left[P_{free}\right]\times \left[S_{free}\right]\times K_{A}$$

(2) φ=φMAX(1+KDc)

(3) $$\Delta G_{binding}=-RTln\left(K_{D}\right)$$

The law of mass action can be rewritten into the Langmuir Equation (see Equation (2)), where the bound density φ can be fitted against the free protein concentration c [18,28]. The Langmuir equation allows for extraction of two central parameters; the maximum density $\varphi_{MAX}$ and the *K_D_*-value. While the driving force of the reaction, in terms of Gibbs free energy, can be calculated directly from the experimentally derived *K_D_*-value (see Equation (3)), the maximum density $\varphi_{MAX}$ can be used to estimate the total number of possible binding sites in a substrate. However, as this model only applies to binding sites that do not change upon binding, it poses problems for proteins that modulate their substrate upon recruitment [28].

### 2.1. Two Insertion Regimes: MCS and MCG

**Figure 3:**
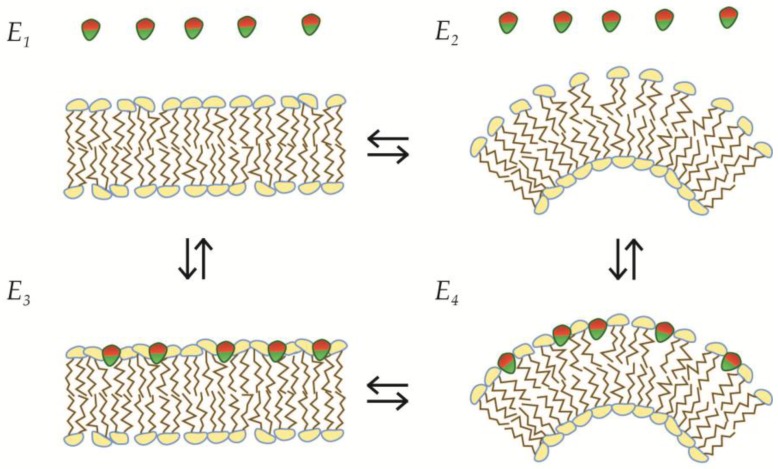
Dual curvature interaction; MCS and MCG by AIMs. The dual nature of curvature stabilizing proteins or motif exemplified with insertion of AIMs, which are depicted with a hydrophobic face (**green**) and a hydrophilic face (**red**).

AIMs are found in a large variety of protein domains, such as the C2 domain, hydrophobic loops, and in particular amphipathic helices [29]. In terms of membrane curvature, AIMs display a dual mode of action, as they both promote recruitment to membranes, while also modulating the morphology of the membrane substrate itself. The ipso facto dual nature of these AIMs can be recognized in an energy-diagram, where the two processes are represented as transformations between specific energy-functions of state (see Figure 3). Because all possible processes can be described as a transition from one energy state to another $\left(E_{x}\to E_{y}\right)$, which can be expressed in terms of the Gibbs free energy change, the direction of a spontaneous process can be predicted through simple considerations. Firstly, since the path between states is arbitrary, the free energy change in a process where a membrane is initially bent and subsequently recruits AIMs is identical to the free energy change of a process where AIMs insert into a flat membrane, and subsequently force it to bend (see Figure 3 and Equation (4)).

(4) $$\begin{array}{c} \Delta G_{x\to y}=G_{x}-G_{y}\\ \mathrm{and}\;\\ \Delta G_{1\to 3\to 4}=\Delta G_{1\to 2\to 4}\\ \mathrm{so}\;\mathrm{if}\;\\ \Delta G_{3\to 4}<\Delta G_{1\to 2}\\ \mathrm{then}\;\\ \Delta G_{2\to 4}<\Delta G_{1\to 3} \end{array}$$

By simple conversion it follows that *if* the transition from a flat membrane to a bent membrane is more favorable when amphiphiles are inserted, *then* the transition from unbound protein and membrane to bound protein on membrane will be more favorable if the membrane is already bent (see Equation (4)). This can be literally formulated in this simple dogma for dual curvature interaction:

*If a forced insertion of an AIM will favor bending of the membrane (MCG), then bending of the membrane will favor insertion of this AIM (MCS)*.

### 2.2. Geometric Models for Insertion of AIMs

**Figure 4:**
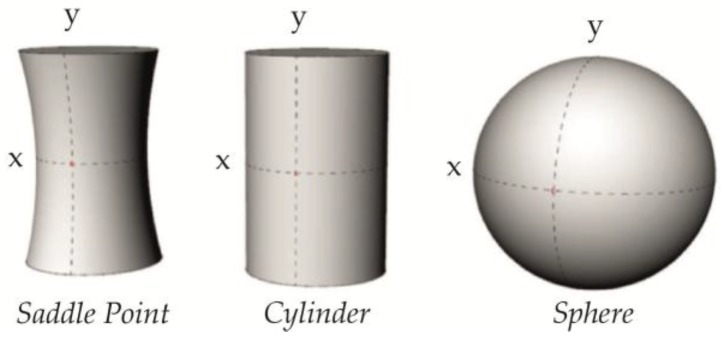
Three cardinal types of geometrical curvature. The geometrical point in each shape is marked with red, where the two principal radii of curvature intersect. The curvature of the *x*-axis in each figure is assumed to be C in each geometrical point, whereas the curvature of the *y*-axis ranges from −C in the saddle-point, over 0 in the cylinder, to +C in the sphere. Hence, the mean curvatures are calculated as 0, 0.5 × C, and C, respectively.

As membranes are two-dimensional structures, interactions between amphiphiles and membrane curvature must be described through two principal radii of curvature, (${ℛ}_{x})$ and (${ℛ}_{y})$, which are defined as the radii of the circle that fits the arc of the curvature best, where $C_{x}=\frac{1}{{ℛ}_{x}}$ and $C_{y}=\frac{1}{{ℛ}_{y}}$. These together distinguish the predicted properties between geometrical shapes (see Figure 4). There are two general types of geometric curvature; mean curvature and Gaussian curvature (see Equation (5)), but as the free energy contribution from Gaussian curvature is mostly negligible, the mean curvature is often referred to simply as the ‘curvature’, and it is this term that will be applied throughout this review.

(5) $$C_{Mean}=\frac{1}{2}\left(\frac{1}{{ℛ}_{x}}+\frac{1}{{ℛ}_{y}}\right);\;C_{Gaussian}=\frac{1}{{ℛ}_{x}}\times \frac{1}{{ℛ}_{y}}$$

During trafficking processes in biological cells, the membranes undergo a large variety of shapes, of which the three cardinal types have been shown in Figure 4: the saddle point (intermediate during scission [4,30]), the cylinder (e.g., Endoplasmatic Reticulum (ER) or recycling tubuli [31,32]), and the sphere (e.g., transport vesicles or lysosomes [6,33]). The cylindrical shapes and the spheres have been experimentally mimicked through various in vitro assays, where cylindrical tubes are pulled from Giant Unilamellar Vesicles (GUVs) and small spherical liposomes are generated directly from lipid films [34,35]. From a geometric point of view, it should be possible to compare properties between these different shapes directly, as a sphere with a 50 nm radius and a tubule with a radius of 25 nm should yield the same effective curvature, and consequently are expected to contain identical intrinsic properties. This approach relies on an assumption of a conserved direct relation between the geometrical curvature and the lipid packing defects in the membrane.

### 2.3. Geometric LPD-Model 1: Saturation of Static Membranes

**Figure 5:**
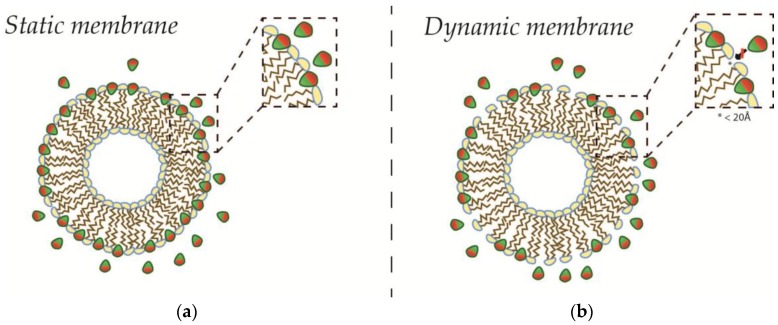
Saturating binding of amphiphiles to static or dynamic membranes. Liposome is shown here with an excess of amphiphiles (**red**/**green**). Inset shows the complete saturation of any putative lipid packing defects on static membranes (**a**); and incomplete saturation with small excess defects below the permissive size for insertion (here 20 Å) on dynamic membranes (**b**).

LPDs provide the “binding sites” for AIMs in the membrane, and the abundance of LPDs have therefore been used as a predictor of possible binding density. In a simple geometric model for MCS, the maximum density $\varphi_{MAX}$ of bound protein was predicted to correlate directly with the calculated sum of de novo LPDs that would arise from the bending of a membrane into a certain curvature (see Equation (6)). The model was originally used to explain MCS phenomena observed in vitro, where the protein binding density scaled continuously with the curvature of liposomes, and the saturation of binding appeared to fit the Langmuir Equation well [18,36]. Hence, the shape of the individual liposome was assumed constant during binding, and the “binding sites” were assumed to be independent (see Figure 5a). Consequently, the maximum density $\varphi_{MAX}$ of bound AIM was predicted to depend directly on the relationship between curvature (1/R), and therefore the relative distribution of defect areas (ΔA/A) and the effective size ($A_{AIM}$) of the AIM (see Equation (6)). When lipids of different effective sizes were recruited to liposomes of different curvatures, this relationship was conferred [18], and this simple geometrical model has since been applied in the Single Liposome Curvature Sensing (SLiC) assay, which uses small unilamellar vesicles (SUVs) as membrane substrate, and where the MCS read-out, an assigned property of the AIM itself, has been the numeric value of the power α [13,37]. However, the simple relation between geometric curvature and AIM recruitment was challenged, as the experiments in GUVs with similar AIMs did not recapitulate the findings with similar lipids [18,25].

(6) $$\varphi_{MAX}\propto \frac{\int \Delta A/A\partial A}{A_{AIM}}=\sum LPD\;\sum LPD\propto {\left(\frac{1}{ℛ}\right)}^{\alpha }\;\alpha \propto MCS$$

### 2.4. Geometric LPD-Model 2: Saturation of Dynamic Membranes

Lipid bilayers are not static substrates of rigid building blocks that are pushed aside when AIMs are inserted, but rather constitute dynamic landscapes in constant change. Therefore, recent models that address the relationship between geometric curvature, as well as lipid composition, and LPDs, often describe the local abundance of LPDs as a statistical distribution. In these models, the abundance of LPDs over an area ($f_{LPD}$) falls off with their LPD-size according to an exponential decay (see Equation (7), upper), where A is the area of the defect and A_C_ is an area constant thought to depend on the lipid composition and geometric shape of the membrane [14,15,16,19]. Putative binding of AIMs into these dynamic membrane systems will reduce the total area of LPDs, which consequently change the distribution of defect areas. Therefore, the size of the expected maximal LPD-size will also be reduced until it reaches a size that is no longer sufficient to accommodate the insertion of additional AIMs (see Figure 5b), and the system will approach saturation (see Equation (7), lower). This is expected to hold true for any rigid AIM.

(7) $$f_{LPD}=e^{\left(-A/A_{c}\right)}\;\underset{f\left(A_{AIM}\right)\to 0}{\lim}\varphi =\varphi_{MAX}$$

Amphipathic helices, however, comprise a special type of AIM in the sense that they usually do not fold into their amphipathic secondary structure before they are fully inserted. The insertion of an amphipathic helix is thus thought to happen through a step-wise process with an initial insertion of one or a few hydrophobic residues, and subsequent folding in the membrane [16]. The potential for further insertion in this case will therefore depend on the prevalence of defects that are big enough to accommodate insertion of such bulky hydrophobic residues, i.e., above 20 Å^2^ [17]. This model integrates the membrane as a dynamic substrate in terms of MCS, rather than assuming independent binding sites, but it does not allow a straightforward prediction of whether or not an insertion is energetically favorable. In addition, the focus of both these geometrical models is isolated on the MCS properties of the AIM, and do not address the putative MCG effect that AIMs might exert on the membranes at saturating conditions. However, such predictions can be made by the combination of these simple geometrical considerations and basic thermodynamics.

## 3. Thermodynamic Insertion-Model

The lipid bilayer is held together by a simple thermodynamic phenomenon known as ‘the hydrophobic effect’, which minimizes the energetically unfavorable interface between the hydrophobic lipid chains and water. This minimization of interface is described through surface tension, a contractive force in the membrane that has been shown to link membrane geometry and the function of transmembrane proteins directly [38]. There are thus no attractive forces between the aliphatic chains per se, but rather a steric repulsion that increases upon membrane contraction. These repulsions add up to a lateral pressure in membranes, in a concentration-dependent manner [39], and the lipid bilayer is consequently held in an equilibrium state, where the expansive forces from the lateral pressure balance the contractive forces of the surface tension [28]. In this aspect, the lipid composition and the local biophysical properties of the membrane are highly interconnected [40,41,42], however, to ease direct comparison with established geometrical models, we assume homogenous and symmetrical lipid compositions across the bilayer throughout this review. Importantly, although the initial spontaneous curvature depends on membrane composition and asymmetries between the leaflets, the changes upon insertion into or bending of the membrane are fundamentally the same from a biophysical point of view.

### 3.1. Basic Intrinsic Properties of Curved Membranes

**Figure 6:**
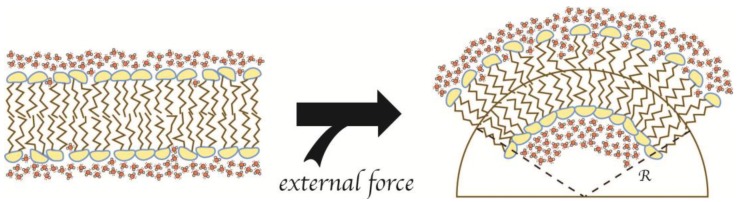
Bending of the lipid bilayer by external forces. The membrane is shown as a lipid bilayer surrounded by water molecules (**red**/**white**). External forces, such as scaffolds, molecular motors, or AIMs, provide the energy to bend the flat membrane (**left**) into a curved morphology (**right**). The outer leaflet of the membrane now increases the interface between the water molecules and the hydrophobic interior of the membrane, while the lipids of the inner leaflet are compressed.

Although lipid bilayers are preferably flat [43], given identical lipid composition on each side, the cell is dominated by membranes of curved morphologies. A wide range of molecular motors, scaffolding proteins, or membrane-inserting proteins drive the formation of these curvatures through MCS/MCG-processes that are governed by the elastic properties of the membrane [17,44,45,46]. When a membrane is bent (see Figure 6), the outer monolayer is displaced from equilibrium by stretching, which is energetically penalized through an increased surface tension (γ), which correlates directly with the relative change of the interface area and therefore also LPDs (see Equation (8), upper). The simultaneous free energy change from decreasing lateral pressure in this monolayer is negligible. Conversely, the inner leaflet is simultaneously displaced from equilibrium by compression and therefore energetically penalized through increased lateral pressure (Π), which correlates with the square of the relative change of the membrane area (see Equation (8), lower). In this case, the simultaneous change of free energy from the decrease in surface tension is also negligible [28,47].

(8) $$\Delta G_{\gamma }\propto \frac{\Delta A_{interface}}{A_{membrane}}\;\Delta G_{\Pi }\propto {\left(\frac{\Delta A_{flat\to bend}}{A_{membrane}}\right)}^{2}$$

Accordingly, the total expected free energy change is estimated as the sum of the energetic penalties in each monolayer (see Equation (9)), which can be directly related to the energy needed to bend the membrane into a certain curvature. When the membrane is described as a thin sheet, these intrinsic elastic properties of the membrane are described directly through the bending modulus ($\kappa_{B}$), the Gaussian modulus ($\kappa_{G}$), and the two types of geometric curvature (see Equation (9)), again, with a negligible contribution from the element containing the Gaussian modulus. It can further be shown that the bending modulus is directly related to the tension and lateral pressure components [28], and since we are focusing on AIMs rather than scaffolding proteins in this review, we examine the recursive effects of binding in terms of these two components instead.

(9) $$\Delta G_{\gamma }+\Delta G_{\Pi }=\Delta G_{total}\;g_{B}\propto \frac{1}{2}\kappa_{B}{\left(C_{x}+C_{y}\right)}^{2}+\kappa_{G}C_{x}C_{y}$$

### 3.2. Saturation of Insertion in Fixed Membrane Morphologies

**Figure 7:**
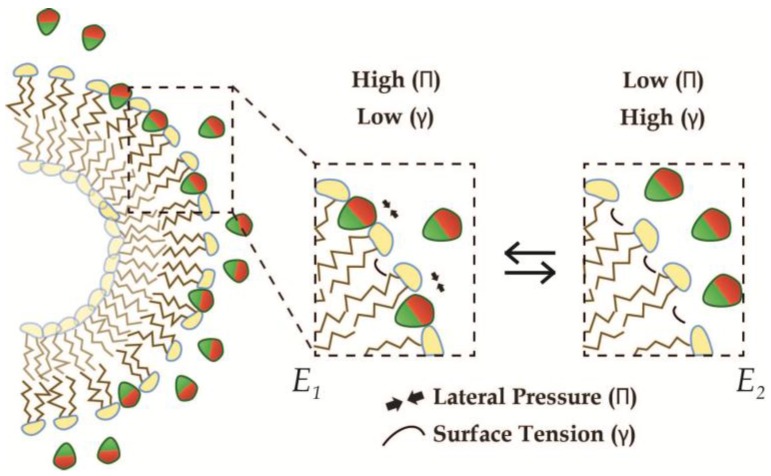
Thermodynamic model of the insertion of AIMs into liposome membranes. Half a liposome is shown here with an excess of amphiphiles (**red**/**green**). Inset shows the insertion of amphiphiles, where the properties of the membranes permit. In energy-state E_2_ (**right**) the outer leaflet is stretched and thereby penalized with an increase in surface tension, while the lateral pressure remains low. Upon amphiphile insertion, the lateral pressure increases, while the surface tension is reduced until further insertion is not energetically favorable (**left**).

If we again consider a membrane held in a curved morphology, e.g., in a liposome, and assume this morphology to be fixed without any flipping of lipids, the insertion of AIMs into the outer leaflet will be energetically driven by the surface tension (see Figure 7). However, as this insertion continues, a counterbalancing increase in lateral pressure in the same leaflet builds up, in a manner corresponding to the effective sizes of the AIMs. Saturation, i.e., maximum insertion density $\varphi_{MAX}$, will then be reached, when the energetic penalty from increasing lateral pressure balances the energetic gain from a surface tension reduction (see Equation (10)).

(10) $$\underset{-\Delta G_{\Pi ,insertion}\to \Delta G_{\gamma ,insertion}}{\lim}\varphi =\varphi_{MAX}\;\varphi_{MAX}\propto A_{AIM}\;\&\;C^{\alpha }$$

Because both the lateral pressure and the surface tension depend directly on a relative change of the membrane surface area (see Equation (9)), the density at saturation will depend on the interplay between the effective size of the AIM and the initial stretching of the outer leaflet, which in turn is related to the simple geometry of the membrane in terms of curvature (see Equation (10)). In a simple isolated system, where the membrane morphology does not change upon interaction, it should thus be possible to derive the MSC and saturation properties directly from the membrane composition and local geometry by relating these factors directly to the properties of the AIM itself. However, as earlier shown, membranes are dynamic substrates that can change morphology upon insertion of AIMs. This phenomenon also holds true for liposomes, which are seen to tubulate, vesiculate, and break in other terms [21,22,23,24].

## 4. Recursive Changes of Membrane Morphology

**Figure 8:**
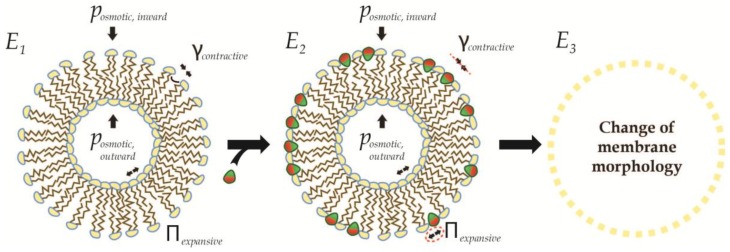
Change of the membrane substrate upon amphiphile insertion. Liposome morphologies are stabilized by opposing forces (**left**), which are generally speaking a combination of contractive forces (surface tension γ and inward osmotic pressure $p_{osmotic,inward}$) and expansive forces (lateral pressure Π and outward osmotic pressure $p_{osmotic,outward})$. Insertion of amphiphiles reduced the surface tension (see red dashed line), while increasing the lateral pressure (see dashed red ellipse), and thereby tips the balance in favor of the expansive forces (**middle**). As a consequence, the membrane will change morphology (**right**).

The size and morphology of a liposome is, in general, held stable by opposing forces; the contractive forces from the surface tension in both monolayers and the inward osmotic pressure, and the expansive forces derived from the lateral pressure in both monolayers, together with the outward osmotic pressure (see Figure 8). AIMs bind only from the outside, which accordingly reduces the surface tension in the outer monolayer, while the lateral pressure is simultaneously increased. This change is asymmetric and disrupts the balancing forces, destabilizes the liposome and concomitantly leads to expansive morphological changes such as swelling or tubulation until a new equilibrium is established (most likely in accordance with the Young-LaPlace relation of capillary pressure [48]). However, since this changed membrane morphology now putatively provides a new substrate for favorable insertion, this insertion/modulation cycle can continue in a recursive manner until further insertion is prevented by restrictive forces or radical change of liposome morphology through breakage or fission.

Derivation of precise models for such pathways are beyond the scope of this review, however we do consider it relevant to discuss the implementation of recursive MCS/MCG-changes, as these are already experienced in vitro, where curvature sensing AIMs promote fission and/or vesiculation of different membrane compartments [24,36,49]. These observations have been recapitulated in cells, where AIMs are not only shown to be important for protein recruitment to curved membrane morphologies, but are also implicated in initial curvature inductions, as well as membrane fission [19,24,50].

### 4.1. Fission in a Closed Uncoupled Compartment

Membrane fission in cells generally happens from tubular extensions, which represent open systems with access to lipid reservoirs. Fission of small closed and uncoupled compartments, e.g., in relation to dense core vesicles during biogenesis, or budding of small vesicles from lysosomes of approximately 500 nm in diameter [33,51], poses other challenges. If we again consider liposomes of membranes without spontaneous curvature, i.e., where the lipid composition is symmetric in between the leaflets, it can be shown that the integrated elastic energy $\left(G_{v}\right)$ over the entire surface is independent of the size, and thereby the curvature, of the vesicle [28] (see Equations (9) and (11)).

(11) $$g_{v}\propto 2\kappa_{B}{\left(\frac{1}{ℛ}\right)}^{2}\;G_{v}=\int g_{v}dA\;G_{v}=8\pi \kappa_{B}$$

**Figure 9:**
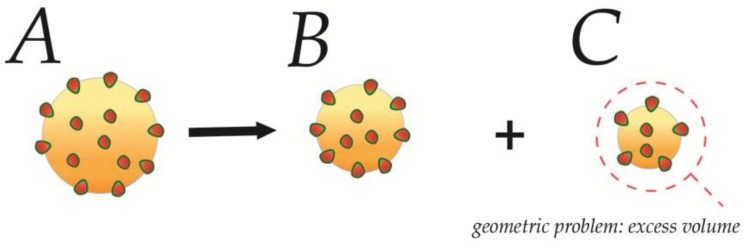
Fission of closed compartments. A closed spherical membrane compartment undergoes fission and generates two smaller compartments. As the integrated elastic energies of **A**–**C** are assumed to be identical, more favorable insertion per surface area are assumed to be permitted on the fission products. However, there will be an excess of volume, which will be expected to destabilize one or both of the fission products (see **red** dashed circle), which will react by changing morphology if energetically favorable.

This implies that the fission of one closed vesicle into two minor vesicles will increase the total elastic energy of the vesicle system, and accordingly allow for more amphiphile insertion (see Figure 9). A similar conclusion can be reached by direct comparison of their respective curvatures, as the two smaller vesicles integrate higher local curvatures over the same summed membrane area (see Equations (10) and (12)). Fission can thus, as expected, be an energetically favorable response to saturating binding.

(12) $$C_{A}>C_{B}>C_{C}\;\varphi_{MAX,A}<\varphi_{MAX,B}<\varphi_{MAX,C}$$

**Figure 10:**
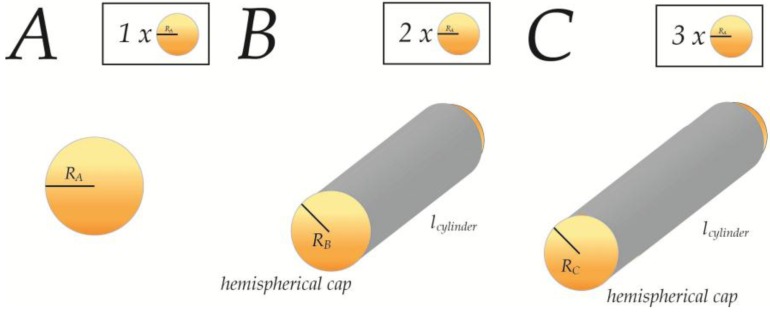
Formation of tubular carriers through homotypic fusion. A spherical membrane compartment **A** undergoes homotypic fusion with another similar spherical compartment, and yields a tubular fusion product **B**, where the radius decreases slightly to accommodate morphological changes as a result of insufficient volume. Fusion with another spherical compartment **A** yields the triple fusion product **C**. Both fusion products are defined as tubes consisting of two hemispherical caps spanned by a cylindrical part.

However, several geometrical problems arise in the case of uncoupled membranes. Firstly, for any fission to occur, the membrane needs to go through a series of unfavorable membrane morphologies [30,52], which becomes highly unlikely without any lipid reservoir to support these changes. Secondly, because the volume scales with ${ℛ}^{3}$, while the membrane area of the liposomes scales with ${ℛ}^{2}$, there will be excess volume $\left(V_{A}>V_{B}+V_{c}\right)$ which cannot be supported by the two resulting liposomes, if we assume conservation of lipid material $\left(A_{A}=A_{B}+A_{c}\right)$ and still negligible leakage of content. In a cellular context, this needs to be considered in relation to the suggested budding of excess membrane material during the maturation of dense core vesicles. In a similar fashion, fusion processes between such closed cellular compartments, as suggested for homotypic fusion of immature secretory granules [53] will yield a fusion product that contains insufficient volume to sustain a sphere, if we still assume no leakage of content (see Appendix A). Instead these fusion-products are forced to take on ellipsoid or tubular shapes, where the surface/volume ratio is higher (see Figure 10). We do recognize that the most likely scenario is the ellipsoid with a more homogenous energy-profile, however choose to continue with tubular shapes for illustration of boundaries and for easier calculation of examples. In such tubular shapes the relative mean curvature $C_{t}$ is increased, relative to its spherical counterpart, due to a reduced radius $r_{t}$ (see Appendix B), and the resulting tubular fusion products will thus retain surprisingly much of their elastic properties in spite of their increase in surface area (see Table 1).

(13) $$\frac{C_{t}}{C_{s}}\propto \frac{r_{t}}{r_{s}}\propto \frac{V_{t}}{V_{s}}$$

(See the exact derivation of $\frac{r_{t}}{r_{s}}$ and $\frac{C_{t}}{C_{s}}$ from $\frac{V_{t}}{V_{s}}$ in Appendix B and Appendix C).

From the table it can be seen that a tubular fusion-product of three identical spheres, will retain 1.43 times the mean curvature, as would the spherical counterpart with sufficient volume. Hence, such fusion-processes can succeed while still maintaining the intrinsic properties, as $\frac{C_{n+1}}{C_{n}}=0.95$. As the intrinsic properties, in terms of membrane tension, are linked to both fission and fusion events [30,52,54], such preservation of properties through geometrical changes could be essential to continuously sustain these processes correctly, or alternatively to continuously recruit the adequate proteins. Interestingly, such tubular shaped carriers have also been observed in the context of fission from plasma membrane, which can be considered both open and coupled, and therefore do not pose the same geometrical challenges [55].

### 4.2. Recursive Morphology Change in Open Coupled Systems

**Figure 11:**
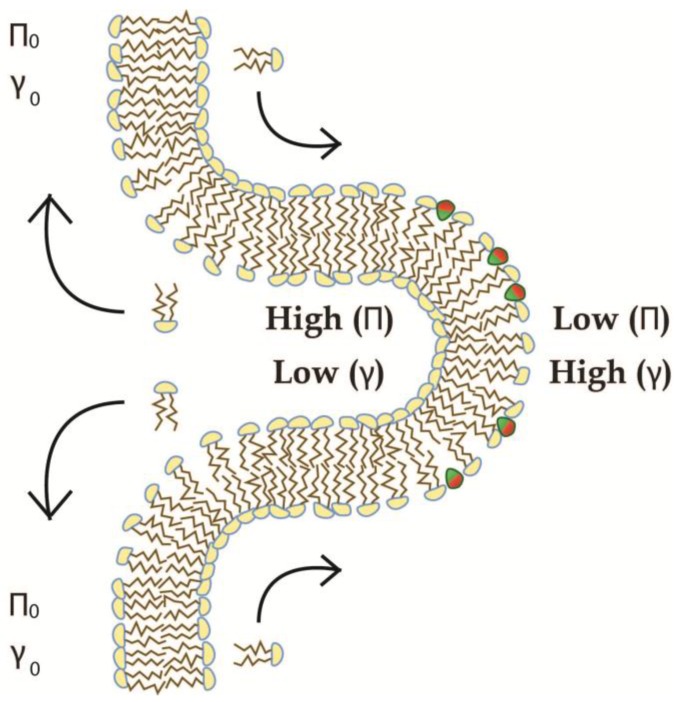
Alterations of properties in local curvatures by the Marangoni Flow of lipids. When local curvatures are generated on coupled membranes, the elastic properties of the local curvature, represented here by surface tension (γ) and lateral pressure (Π) are changed relative to the elastic properties of the membrane reservoir ($\gamma_{0}\;\mathrm{and}\;\Pi_{0})$. This will generate a flow of lipid away from the area of high lateral pressure into the area of higher surface tension (arrows).

When local curvatures are generated on larger lipid reservoirs, there will be a fast lateral flow of lipids [25], and the free diffusion of water will keep the volume as well as the osmotic pressure stable during any morphology changes. Thus, an expected surface tension penalty in the outer monolayer will be relaxed by a flow of lipids into the region of local curvature, while the energetic penalty from lateral pressure in the inner leaflet will be correspondingly reduced by a flow of lipids out of the local curvature (see Figure 11). The lateral flow of AIMs (e.g., lipids) from a region of low surface tension to a region of high surface tension is called a Marangoni-Flow and the extent of the flow (ℝ) has been shown to correlate directly with the change of surface tension $\left(\Delta \gamma_{local\;vs.reservoir}\right)$ between the reservoir and local regions [56] (see Equation (14)).

As the relaxation of elastic energy in the local curvature will relate directly to the relative change of area, as earlier shown, the relaxation by Marangoni Flow ($\partial A_{Marangoni}$) will be directly related to the effective size of the AIM and the number of AIMs flowing to the local curvature ($\Delta N_{AIM}$), which in turn is directly related to the difference in surface tension (see Equations (14) and (15)). As the difference in surface tension is directly linked to the relative morphology-change at the local curvature, we therefore predict it will change in a continuous manner according to the “degree of reservoir coupling” (DRC).

(14) $$\mathbb{R}\propto \Delta \gamma_{local\;vs.reservoir}$$

(15) $$\partial A_{Marangoni}=A_{AIM}\times \Delta N_{AIM}\;\Delta N_{amph}\propto \mathbb{R}\propto \Delta \gamma_{local\;vs.reservoir}\;\Delta \gamma \propto \frac{C_{reservoir}-C_{local\;curvature}}{C_{local\;curvature}}=DRC$$

In the earlier case of homotypic fusion, where closed compartments are forced into tubular shapes, due to an insufficient volume to sustain a sphere, the overall mean curvature was relatively preserved by structural changes into tubular shapes. However, by simple geometrical considerations the hemispherical ends are expected to contain very high local curvatures in these tubular shapes (see Figure 10). However, as a consequence of the predicted Marangoni Flow, even such high apparent local curvatures of the hemispheres would be diminished by flow from the cylindrical part, which take up to 83.4% of the tube in fusion product C (see Appendix B for calculations). The relatively lower fraction of cylindrical geometry in fusion product B (73.2%) would relax the local tension to a relatively lesser extent, and no unidirectional flow would be expected in sphere A, where the surface is assumed homogenous for this review. Ultimately, the geometrical restraints and DRC both constitute mechanisms by which recursive changes in membrane morphology can counteract local changes in intrinsic properties, e.g., in the process of homotypic fusion.

In summary, an area constant (see Equation (7)), which defines the distribution of expected LPDs ($A_{c}$), will be expected to depend not only on lipid composition $\left(A_{c,comp}\right)$ and local membrane geometry $\left(A_{c,geom.}\right)$, but also contain a component of DRC $\left(A_{c,DRC}\right)$ (see Equation (16)). In thermodynamic terminology, the actual free energy change of insertion into a coupled membrane will be numerically lower than if calculated directly from lipid composition and local geometry ($\Delta G_{comp,geom}$) in a manner that relates directly to the relative properties of the reservoir (see Equation (17)).

(16) $$A_{c}=A_{c,comp}+A_{c,geom.}+A_{c,DRC}$$

(17) $$\Delta G_{insertion}=\Delta G_{comp,geom.}+\Delta G_{DRC}$$

## 5. Summary

In summary, based on a review of the current models that describe the interaction of amphiphilic insertion motifs (AIM) with membranes, we employ simple thermodynamic considerations to extend the current models to consider the membrane as a dynamic entity. This enables a description of the recursive nature of AIM-membrane interactions and predicts the degree of reservoir coupling as a key determinant of membrane biophysical properties, with implications for AIM-membrane interactions as well as membrane fusion and fission processes.

## 6. Discussion

**Figure 12:**
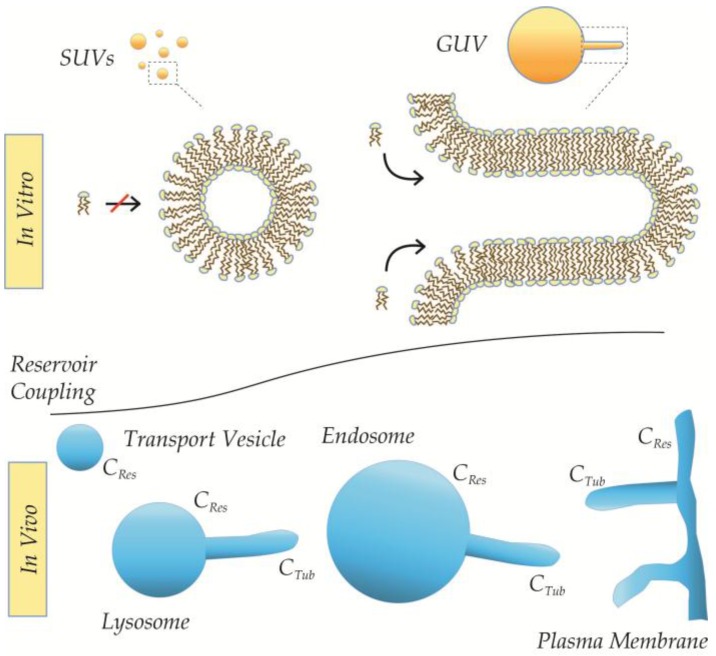
Effect of DRC in vitro and in vivo. Local curvatures of identical lipid compositions and local geometries will differ in intrinsic properties in vitro, where the local curvature on the tube has higher coupling than a similar local curvature on a SUV (**upper**). Likewise, will the properties of similar local curvatures on tubes pulled from lysosomes, endosomes, and plasma membrane differ in respect to the size and shape of their lipid reservoirs (**lower**). Importantly, these differences likely will carry over in resulting tubular and vesicular carriers.

The exact derivations of constants and free energy relations are beyond the scope of this review, but we speculate that the effect of DRC might underlie some of the reported discrepancies between the apparent MCS of lipids in SUV-systems and the lack of MCS by similar lipids in the GUV-system [18,25] (see Figure 11 and Figure 12). Moreover, the DRC is likely to account at least in part for the more pronounced role of AIMs in membrane curvature sensing and generation observed on liposomes as compared to tubules of the same curvature (*C*_mean_) [25,36,57]. The model further predicts differences in the nature of budding of isolated carriers from closed compartments (e.g., sorting from secretory vesicles) as opposed to continuous membranes (e.g., endocytosis). Equally important, however, is the prediction that the membrane identity of carriers, in terms of morphology and elastic properties, in turn will reflect their individual history of generation. Consequently, we suggest that the DRC of local curvatures should be considered at equal terms with classic determinants as lipid composition and geometry when assessing recruitment properties as well as fusion/fission propensities of the membrane (see Figure 12).

## Figures and Tables

**Table 1:** Preservation of mean curvature in tubular carriers of less volume.

Relative Change	A (1 × A)	B (2 × A)	C (3 × A)
$V_{t}/V_{s}$	1	$\frac{1}{\sqrt{2}}$	$\frac{1}{\sqrt{3}}$
$C_{n+1}/C_{n}$	NaN	0.87	0.95
$C_{t}/C_{s}$	1	1.22	1.43

$V_{t}/V_{s}$ is the relative reduction of volume between the theoretical spherical volume to sustain the surface area of the fusion product (V_s_) and the actual volume in the tubular fusion product (V_t_); $C_{n+1}/C_{n}$ is the relative change of mean curvature between the fusion product ($C_{n+1}$) and its predecessor ($C_{n+1}$); $C_{t}/C_{s}$ represents the relative change of curvature in the fusion product in response to the relative reduction of volume $V_{t}/V_{s}$.

## Appendix A

Relative reduction in volume through homotypic fusion:

(A1) A+A=>B

surface area (*A*) is conserved, i.e.,:

(A2) $$2\times A_{A,sphere}=A_{B,sphere}$$

if the area constitutes a sphere, and *R_B,sphere_* represents that hypothetical sphere, then:

(A3) $$R_{B,sphere}=\sqrt{2}\times R_{A,sphere}$$

volume-input is calculated as the sum of volumes from the two spheres:

(A4) $$V_{2\times A}=2\times \left(\frac{4}{3}\times \pi \times R_{A,sphere}^{3}\right)$$

volume needed to sustain the hypothetical sphere made up from the summed surface areas:

(A5) $$V_{B,sphere}=\frac{4}{3}\times \pi \times {\left(\sqrt{2}\times R_{A,sphere}\right)}^{3}$$

by substitution is obtained:

(A6) $$V_{B,sphere}=\sqrt{2}\times V_{2\times A}$$

where after the relative reduction in volume can be calculated:

(A7) $$\frac{V_{2\times A}}{V_{B,sphere}}=\frac{V_{t}}{V_{s}}=\frac{1}{\sqrt{2}}$$

## Appendix B

Derivation of tube radius $r_{t}$ from the relative reduction in volume $\frac{V_{t}}{V_{s}}$:

Area $A_{s}$ and Volume $V_{s}$ of the hypothetical sphere:

(A8) $$A_{s}=4\times \pi \times r_{s}^{2}$$

(A9) $$V_{s}=\frac{4}{3}\times \pi \times r_{s}^{3}$$

Though a reduced volume most likely would produce an ellipsoid geometry, we here assume a complete conversion into a tubular carrier, which makes the calculations more approachable.

Area $A_{t}$ and Volume $V_{t}$ of the tubular product with reduced volume:

(A10) $$A_{t}=4\times \pi \times r_{t}^{2}+2\times \pi \times r_{t}\times l_{c}$$

(A11) $$V_{t}=\frac{4}{3}\times \pi \times r_{t}^{3}+\pi \times r_{t}^{2}\times l_{c}$$

where $l_{c}$ represents the length of the cylinder that separates the two hemispherical caps.

As the surface area is maintained, the cylinder length can be expressed in terms of the respective radii:

(A12) $$\frac{A_{t}}{A_{s}}=\frac{4\times \pi \times r_{t}^{2}+2\times \pi \times r_{t}\times l_{c}}{4\times \pi \times r_{s}^{2}}=1⟺\frac{r_{s}^{2}-r_{t}^{2}}{0.5\times r_{t}}=l_{c}$$

hence, any reduction in volume can predict the relationship between the radius of the hypothetical sphere and the actual radius of the tube:

(A13) $$\frac{V_{t}}{V_{s}}=-\frac{1}{2}\times {\left(\frac{r_{t}}{r_{s}}\right)}^{3}+\frac{3}{2}\times \left(\frac{r_{t}}{r_{s}}\right)$$

which can be converted to a third degree polynomial and solved in terms of $\frac{r_{t}}{r_{s}}$ and $\frac{V_{t}}{V_{s}}$:

(A14) $${\frac{r_{t}}{r_{s}}}^{3}-3\frac{r_{t}}{r_{s}}+2\frac{V_{t}}{V_{s}}=0$$

because $0<\frac{V_{t}}{V_{s}}<1$, the solution can be shown to be (see Appendix C):

(A15) $$\left(\frac{r_{t}}{r_{s}}\right)=-0.5\left(1+i\sqrt{3}\right)\sqrt[{3}]{\sqrt{{\frac{V_{t}}{V_{s}}}^{2}-1}-\frac{V_{t}}{V_{s}}}-0.5\left(1-i\sqrt{3}\right)\frac{1}{\sqrt[{3}]{\sqrt{{\frac{V_{t}}{V_{s}}}^{2}-1}-\frac{V_{t}}{V_{s}}}}$$

When the radius of the hypothetical sphere $r_{s}$ is calculated, the radius $r_{t}$ of the tube can be directly derived.

The mean curvature of the hypothetical sphere $C_{s}$ is calculated directly as: $\frac{1}{r_{s}}$, whereas the mean curvature of the resulting tube $C_{t}$ is a function of respective curvatures of cylindrical $C_{cyl}$ and hemispherical $C_{hs}$ parts, respectively, weighted with the fraction θ they represent of the total surface area, e.g., $\left(\theta_{hs}=\frac{A_{hs}}{A_{hs}+A_{cyl}}\right)$:

(A16) $$C_{t}=\theta_{hs}\times C_{hs}+\theta_{cyl}\times C_{cyl}$$

## Appendix C

The third degree polynomial from Appendix B;

(A17) $${\frac{r_{t}}{r_{s}}}^{3}-3\frac{r_{t}}{r_{s}}+2\frac{V_{t}}{V_{s}}=0$$

is written as

(A18) $$x^{3}-3x+2d=0,\text{ where }d\text{ is treated as a constant}$$

making the substitution: x=y+1/y, which yields an equation of the form:

(A19) $$y^{3}+{\left(\frac{1}{y}\right)}^{3}+d=0$$

multiplying with $y^{3}$ and making the substitution: $z=y^{3}$, yields a second order equation:

(A20) $$z^{2}+2dz+1=0$$

let u and v denote the two solutions of this second order polynomial, then:

(A21) $$u=-d+\sqrt{d^{2}-1}\;v=-d-\sqrt{d^{2}-1}$$

as these solutions, by construction, are solutions to $y^{3}+{\left(\frac{1}{y}\right)}^{3}+d=0$, this polynomial has solutions:

(A22) $$y=\sqrt[{3}]{-d\pm \sqrt{d^{2}-1}}$$

substituting this solution back, gives these following solutions to *x*:

(A23) $$x=\sqrt[{3}]{-d\pm \sqrt{d^{2}-1}}+\frac{1}{\sqrt[{3}]{-d\pm \sqrt{d^{2}-1}}}$$

(A24) $$=-0.5\left(1+i\sqrt{3}\right)\sqrt[{3}]{\sqrt{d^{2}-1}-d}-0.5\left(1-i\sqrt{3}\right)\frac{1}{\sqrt[{3}]{\sqrt{d^{2}-1}-d}}$$

and

(A25) $$x=-0.5\left(1-i\sqrt{3}\right)\sqrt[{3}]{\sqrt{d^{2}-1}-d}-0.5\left(1+i\sqrt{3}\right)\frac{1}{\sqrt[{3}]{\sqrt{d^{2}-1}-d}}$$
